# A Rare Occurrence of Chylopericardium in a Case of Anterior Mediastinal Mass

**DOI:** 10.7759/cureus.62214

**Published:** 2024-06-12

**Authors:** Nadhu Krishna Maheswaran Nair, Richu Bob Kurian, Balamugesh Thangakunam

**Affiliations:** 1 Pulmonary Medicine, Christian Medical College Vellore, Vellore, IND

**Keywords:** cardiac tamponade, chylothorax, chylopericardium, adenocarcinoma, anterior mediastinal mass

## Abstract

A middle-aged gentleman presented with dyspnea and hypotension, accompanied by an anterior mediastinal mass infiltrating the anterior chest wall and a moderate to large pericardial effusion, which upon evaluation revealed chylous fluid. Further investigation diagnosed him with right lung adenocarcinoma infiltrating the chest wall, staged at T4N3M1. The patient subsequently developed cardiac tamponade, necessitating immediate medical intervention.

Management of the patient's cardiac tamponade involved pericardiocentesis via an indwelling pericardial catheter, allowing continuous drainage of the chylous fluid. Additionally, the patient was placed on a medium-chain triglyceride diet (MCTD) to reduce chyle production. These interventions resulted in significant symptomatic improvement, stabilizing the patient's hemodynamic status, and alleviating the immediate life-threatening condition.

This case highlights the clinical challenges posed by rare presentations such as chylopericardium secondary to malignancy and emphasizes the importance of comprehensive diagnostic evaluation and prompt therapeutic management. The successful outcome, achieved through a combination of pericardial drainage and dietary modifications, underscores the critical role of a conservative approach in managing complex oncological cases with acute complications.

## Introduction

Chylopericardium primarily involves the accumulation of chylous fluid within the pericardial sac. Hasebroek first described this uncommon finding in the literature [1]. In a normal adult human, between 15 to 50 ml of pericardial fluid is typically present [2]. Chylopericardium is generally uncommon but can occur following cardiothoracic surgery, trauma, tumors, or infections. When the etiology is unknown, it is referred to as primary or idiopathic chylopericardium. The occurrence of chylopericardium in a case of anterior mediastinal wall adenocarcinoma is a rare entity, and the presence of shock and tamponade at presentation is an uncommon finding.

## Case presentation

A 55-year-old patient with a history of three months of dyspnea, cough, and chest pain presented to our emergency department with five days of acute worsening of symptoms. Initial evaluation revealed Type 1 respiratory failure with hypotension, with blood pressure around 80/60 mm Hg and a pulse rate of 120 bpm. Physical examination revealed a 15 cm x 15 cm firm mass on the anterior chest wall. There was puffiness of the face and distended superficial veins on the anterior chest wall, indicating superior vena cava obstruction. Auscultation revealed muffled heart sounds and absent breath sounds in the left infrascapular area. Inotropic support with noradrenaline and high-flow supplemental oxygen via a non-rebreather mask was initiated, and the patient was transferred to the ward.

**Figure 1:**
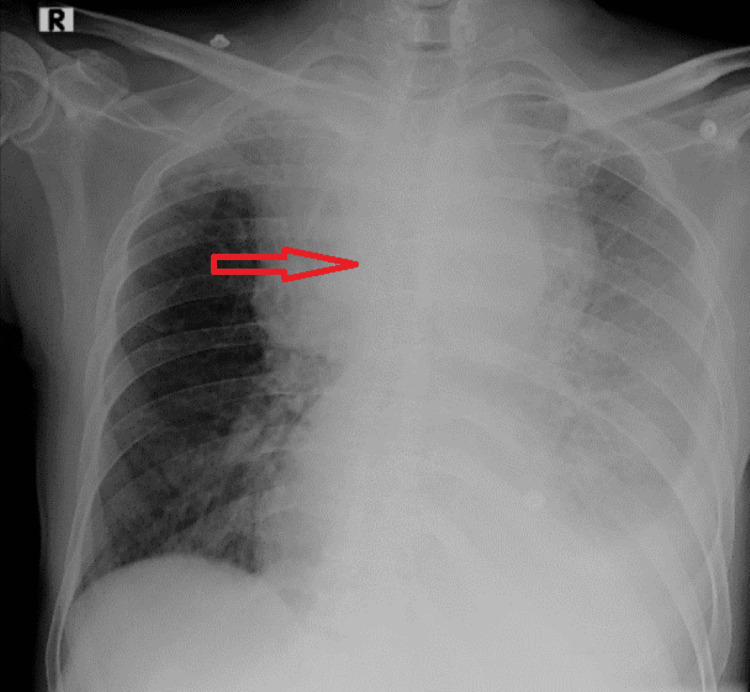
Chest X-ray showing widened mediastinum and left sided pleural effusion.

Initial blood investigations showed normal counts, along with normal liver and renal parameters. The patient's past indicates a 40-year history of smoking with a smoking index of 200 and no other addictions or significant medical conditions. There is no reported family history of malignancy. A chest X-ray (Figure 1) showed a widened mediastinum with a moderate left-sided pleural effusion. Ultrasound-guided diagnostic and therapeutic thoracocentesis was performed, revealing milky-colored fluid. Analysis of the pleural fluid showed a lymphocytic-predominant exudate with low adenosine deaminase values and cytology negative for malignant cells. Acid-fast bacilli smear, Xpert tuberculosis (TB) polymerase chain reaction (PCR), Mycobacterial Growth Indicator Tube (MGIT) culture, and routine bacterial culture were negative. Pleural fluid triglycerides were elevated (192 mg%) and cholesterol was 60 mg%, suggestive of chylothorax.

**Figure 2:**
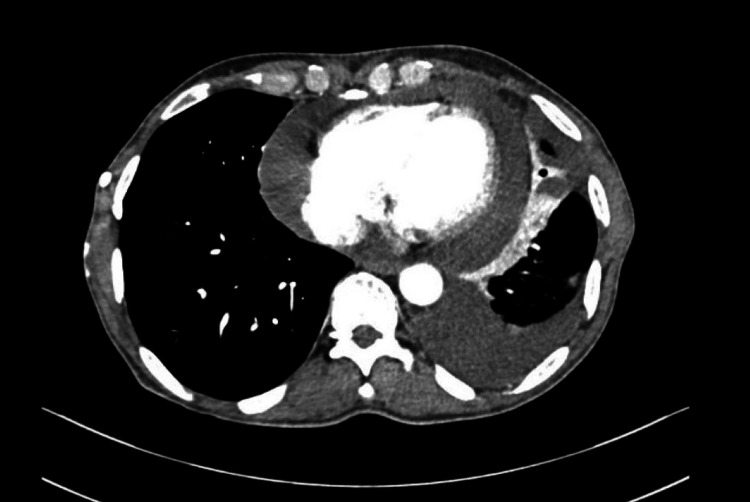
Contrast-enhanced CT thorax showing large pericardial effusion with a left-sided pleural effusion.

**Figure 3:**
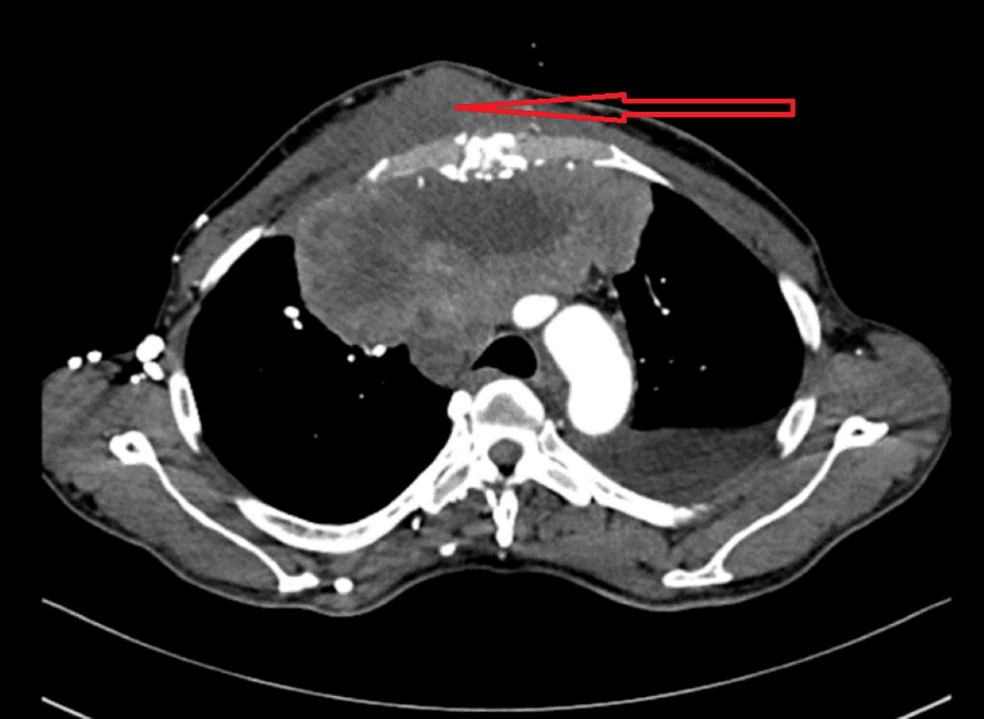
Contrast-enhanced CT thorax showing anterior mediastinal mass infiltrating the chest wall.

**Figure 4:**
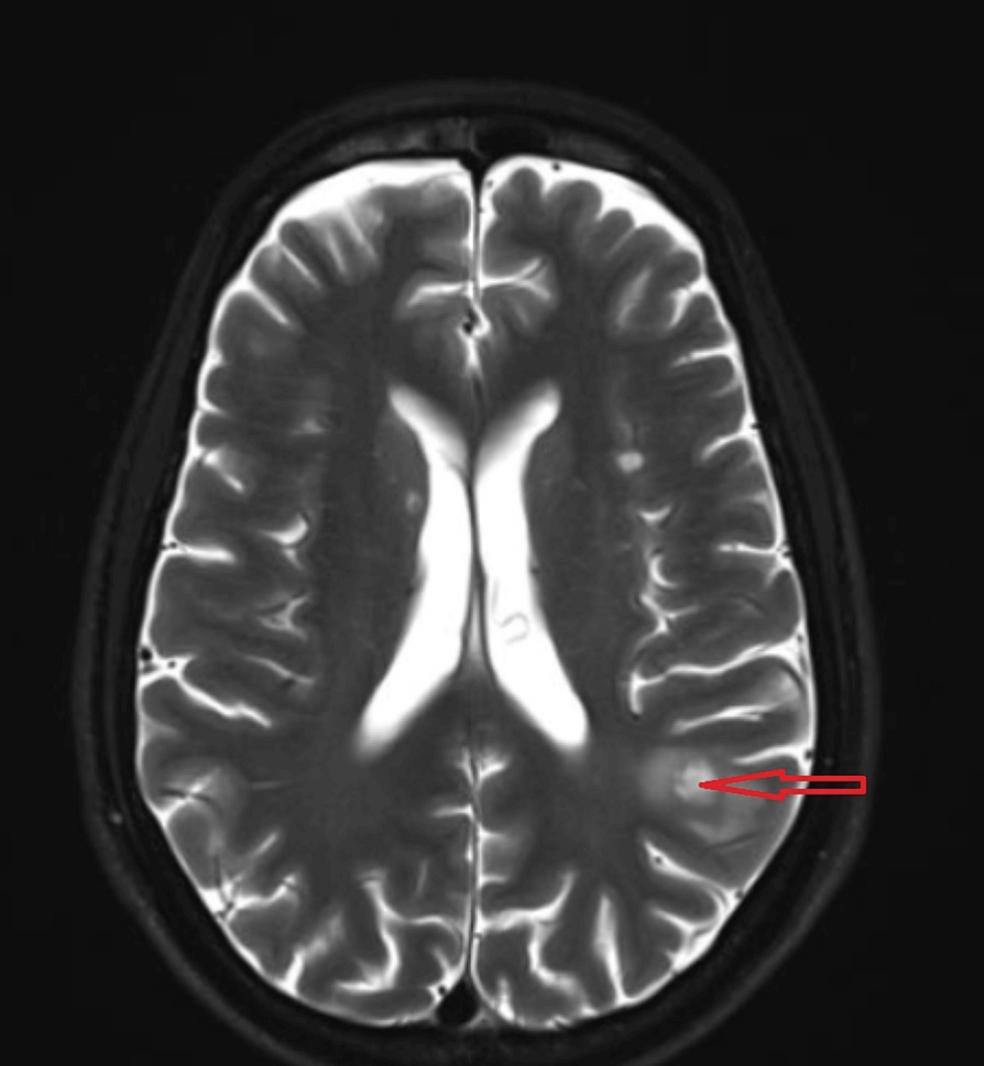
MRI of the brain showing ring-enhancing lesions in the brain parenchyma.

A 12-lead electrocardiogram showed sinus rhythm with low-voltage complexes. Echocardiography revealed evidence of moderate to large pericardial effusion with pulmonary hypertension and right ventricular diastolic collapse. Contrast-enhanced computed tomography (CECT) revealed a large pericardial effusion (Figure 2) and a heterogeneously enhancing ill-defined lesion with solid and necrotic components measuring 13.8 x 11 x 10.5 cm in the anterior mediastinum, infiltrating the anterior chest wall (Figure 3). There was tumor invasion and obstruction of the superior vena cava (SVC), as well as evidence of lymphatic duct obstruction. MRI of the brain (Figure 4) showed multiple ring-enhancing lesions in the brain parenchyma, consistent with metastatic lesions.

An ultrasound-guided biopsy of the anterior chest wall mass revealed adenocarcinoma of the right lung extending to the anterior chest wall (T4N3M1). EGFR, ALK, and PD-L1 mutation analyses were negative.

**Figure 5:**
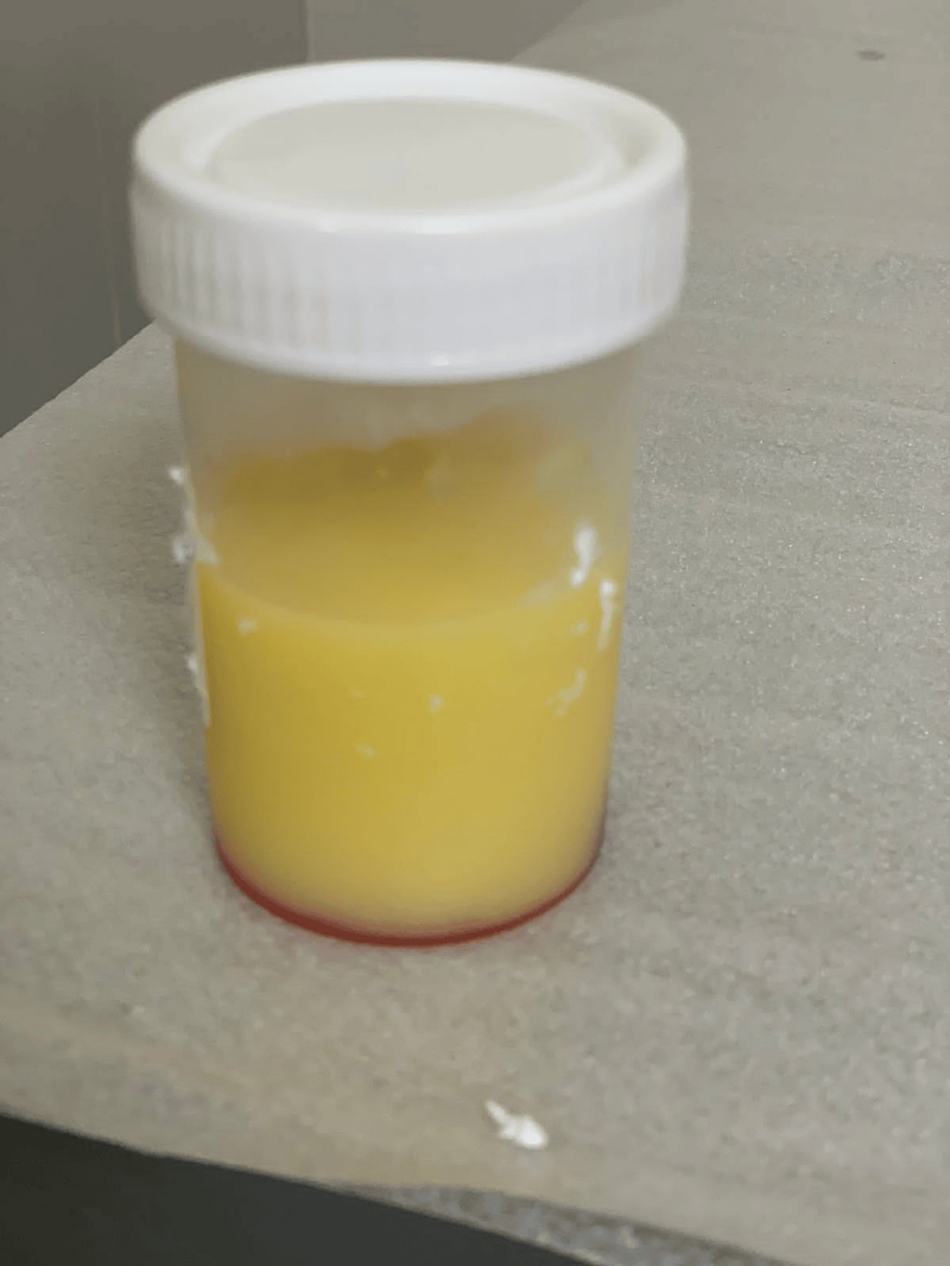
Chylous pericardial fluid.

Diagnostic and therapeutic pericardiocentesis was performed, and 300 ml of milky fluid (Figure 5) was drained. Pericardial fluid analysis revealed triglyceride levels of 631 mg% and a total cholesterol value of 74 mg%. Culture, Xpert TB PCR, and MGIT culture were negative. A diagnosis of chylopericardium with chylothorax was made. The cytology of the pericardial fluid showed atypical polygonal cells with moderate nuclear pleomorphism, dense chromatin, some with visible nucleoli, and moderate amounts of eosinophilic cytoplasm. The atypical cells were highlighted by BerEP4, and mesothelial cells were highlighted by calretinin, suggesting malignancy.

The patient was initiated on a low-fat, medium-chain triglyceride (MCT) diet. An indwelling pericardial catheter was placed for repeated aspiration. Twice-daily aspiration of the chylopericardium was performed, resulting in improved blood pressure; consequently, inotropes were tapered and stopped. Oxygenation also improved accordingly. The patient opted against palliative chemotherapy and was discharged at his request with minimal supplemental oxygen. Further follow-up with the patient was hindered due to communication barriers.

## Discussion

The thoracic duct originates in the abdomen from the sac called the cisterna chyli at the level of the second lumbar vertebrae and passes through the aortic diaphragmatic hiatus to the posterior mediastinum on the right side of the thorax. It crosses over to the left side at the level of the fifth thoracic vertebrae and drains into the left subclavian-brachiocephalic venous junction [3]. Any obstruction in the normal lymph flow or elevated pressure within the thorax can cause a positive pressure in the lymphatic drainage, which can lead to the accumulation of chylous fluid within the pericardium [4]. In our case, an anterior mediastinal adenocarcinoma caused a lymphatic duct obstruction, which forced the chyle to accumulate within the pericardial and pleural spaces. The invasion of lymphatic channels by the carcinoma can also lead to lymph stasis, possibly resulting in chylothorax and chylopericardium.

The first instance of isolated chylopericardium was reported in 1954 by Groves and Effler [5]. Till 1992, Akamatsu et al. reported about 79 cases, and a total of 89 cases were reported by Yuksel et al. till 1997 [6]. Dib et al. have provided insight into 33 reported cases of chylopericardium from 1996 to 2006 [7]. Through a systematic literature review, we found an additional 40 cases of chylopericardium after 2006, taking the total number of chylopericardium cases reported worldwide to 162. Among these 40 cases, there is an equal distribution in the etiology of chylopericardium between tumor-related, idiopathic, and iatrogenic chylopericardium. Of these, only three cases were reported as chylopericardium, presenting as SVC syndrome [8].

Routine image-guided diagnosis of chylopericardium is difficult due to the absence of pertinent diagnostic features. A chest X-ray can show an enlarged cardiac silhouette, and echocardiography can reveal pericardial effusion. The diagnosis of chylous pericardial effusion is established when pericardial fluid shows a triglyceride level of more than 500 mg% and a total cholesterol-to-triglyceride ratio of less than one [7]. Our case had a total pericardial fluid cholesterol of 74 mg% and a triglyceride value of 631 mg%, which was consistent with the diagnosis. Nuclear medicine imaging studies of the lymphatic vessels, such as lymphoscintigraphy, are being increasingly used in the diagnosis of chylopericardium. Other modalities include lymphangiograms and lipiodol injections, which could have therapeutic properties as well by occluding the thoracic duct [9]. Routine imaging supplemented by pericardial fluid analysis was used in our case to obtain a definitive diagnosis. Cytological analysis showed a lymphocytic predominant picture with a sterile culture, as in other reports [4]. However, our cytology report also revealed a few atypical cells suggestive of malignancy, which suggests that chylopericardium need not always have negative cytology. Prompt diagnosis is crucial because the patient may present with symptoms of cardiogenic shock, as was the case in our case.

There is no universally acceptable management plan as there are multiple clinical and etiological factors associated with each case, which warrants a case-by-case approach. The initial treatment plan depends on the prevalence of shock or cardiac tamponade. According to European Society of Cardiology guidelines, patients with pericardial effusion and evidence of hemodynamic compromise should undergo urgent drainage of the pericardial effusion for therapeutic and diagnostic purposes [10]. In our case, the patient presented with features of cardiogenic shock, and a plan for continuous drainage with the help of an indwelling pericardial catheter was made [11].

With adequate drainage, hypoxia and hypotension improved, and the patient experienced symptomatic relief. A subsequent decrease in daily drain output to 150 ml on the second day prompted a literature review, and we decided to continue a conservative approach with drainage and dietary modifications [4]. A low-fat diet with medium-chain triglycerides (MTCD diet) was chosen from among other options that included only total parenteral nutrition (TPN) or both TPN and MCTD diets [7]. Chan et al. show that the conservative approach is successful only in 55 percent of the cases [12]. However, in conditions with large recurrent chylous effusions and significant nutritional loss, a surgical approach involving ligation of the thoracic duct and tributary lymphatics or the creation of a pericardio-peritoneal shunt is advised [13,14].

## Conclusions

In conclusion, the present study reported a case of hypoxia with hemodynamic shock due to chylopericardium, secondary to an anterior mediastinal tumor obstructing the lymphatic drainage. Pericardiocentesis provided an exact diagnosis, and conservative management with dietary modifications and therapeutic drainage was found to be effective.
